# A Meta-Analysis of Global Prevalence of Psittacine Beak and Feather Disease Virus Infection and Associated Risk Factors

**DOI:** 10.3390/ani15101473

**Published:** 2025-05-20

**Authors:** Xueping Zhang, Hongxiang Liu, Jiayu Shi, Hongyu Zhou, Xinyi Lin, Huiling Zhang, Tangjie Zhang

**Affiliations:** 1 Institute of Comparative Medicine, College of Veterinary Medicine, Yangzhou University, Yangzhou 225009, China; zhangxp1995@foxmail.com (X.Z.); 18251238559@163.com (J.S.); zhou2096177403@foxmail.com (H.Z.); mzdzh@foxmail.com (X.L.); 2 Jiangsu Co-Innovation Center for Prevention and Control of Important Animal Infectious Diseases and Zoonoses, Yangzhou 225009, China; 3 Jiangsu Institute of Poultry Sciences, Yangzhou 225009, China; lhxatyz@foxmail.com; 4 Independent Researcher, New York, NY 11355, USA

**Keywords:** parrots, psittacine beak and feather disease virus, prevalence rate, meta-analysis

## Abstract

Beak and feather disease virus is a member of the family *Circoviridae*, and among birds, parrots are susceptible hosts. This study reported the global molecular prevalence and risk factors of BFDV infection in parrot species. Relevant studies were identified from PubMed, Scopus, Web of Science, China National Knowledge Infrastructure (CNKI), and WanFang databases. We retrieved 30 studies encompassing 16,901 parrots from 30 species across six continents and 34 countries, all published between 2003 and 2024. The results showed that the global molecular prevalence of BFDV, based on eligible areas, was 16.30% (95% CI, 11.40–22.00%) using a random-effects model. Subgroup analysis showed that there were significant differences among genera, with *Agapornis* having the highest prevalence of 26.60% (95% CI, 9.80–46.50%). There were significant differences in prevalence rates among the four sampling methods (*p* < 0.01) (blood, cloacal swabs, feather, and fecal). Blood samples exhibited the lowest prevalence at 11.2% (95% CI: 4.4–20.7%), suggesting that BFDV prevalence may be underestimated if only blood tests are used. In regions with distinct seasonal variation, the prevalence of BFDV (3.80%, 95% CI: 0.10–11.10%) was notably lower in summer, but higher in spring and autumn. Age subgroup prevalence varied significantly, with young birds having the highest prevalence at 22.5% (95% CI, 8.8–39.6%). No significant differences in the prevalence of BFDV were observed based on sex or sampling time. This study indicated that the global molecular prevalence of BFDV infection has increased in recent years, and that species, season, sampling methods and age are the main risk factors. By monitoring BFDV prevalence, identifying high-risk species, and elucidating key risk factors, we can develop targeted management strategies to mitigate viral transmission.

## Simple Summary

In this study, we conducted the first meta-analysis and systematic review of the global epidemiology of psittacine beak and feather disease virus (PBFDV) and its associated risk factors. A total of 30 selected studies employing molecular detection methods were included in the analysis. The global prevalence of BFDV in eligible areas was estimated at 16.30%, with the highest infection rate observed in *Agapornis* species, which may be associated with their popularity in the growing pet bird trade. Age susceptibility analysis revealed that young birds exhibited the highest infection rates, followed by nestlings. Geographical variations in infection rates were also identified, with higher prevalence observed in Asia and Africa. In regions with marked seasonal variation, the prevalence of BFDV was significantly lower during the summer. Relying solely on blood samples for detection may underestimate the true prevalence of BFDV. To mitigate cross-regional and cross-species transmission of BFDV, continuous surveillance and monitoring of the virus remain imperative during high-risk periods.

## 1. Introduction

Beak and feather disease virus (BFDV) causes psittacine beak and feather disease (PBFD), a highly contagious disease among wild and captive populations of birds. PBFD is characterized by the appearance of shriveled, brittle and discoloured feathers, as well as deformities of certain parrot beaks and claws [1]. BFDV is a non-enveloped DNA virus of the genus *Circovirus* in the family *Circoviridae*. It has an icosahedral capsid and a small genome (1.7–2.0 kb) that encodes only two proteins [2]. Circoviruses are extremely stable in vitro and are resistant to harsh external environments, high temperatures, and chemical reagents [3]. In addition, because circovirus infection can cause immunosuppression, infected animals might be susceptible to secondary infections with other pathogenic microorganisms, such as bacteria, fungi, and mycoplasmas [4].

BFDV can be transmitted horizontally and vertically, with horizontal transmission being relatively more common and severe [5,6]. Amery-Gale et al. [7] reported the high prevalence (56.2%) of PBFD in psittacine birds in Australia. All species throughout the order *Psittaciformes* are thought to be susceptible to BFDV. Initially identified in wild parrots in Australia [8], PBFD has spread globally, largely driven by the booming international trade in exotic pets and viral mutations. From 2006 to 2012, birds were reported to be the most species-rich and abundant group in trade [9]. Moreover, an increasing number of people are choosing to keep parrots as companion pets, with at least 259 parrot species being owned currently worldwide, and the booming trade in ornamental birds such as parrots has facilitated the spread of BFDV [10,11,12,13].

To date, BFDV has spread around the world and has been detected in more than 60 species of parrots, representing more than 10% of parrot species [12,14]. BFDV is a particular threat to threatened parrots and other birds worldwide in the context of continuing biodiversity decline [15].

An increasing amount of epidemiological data is providing a stronger evidence base for studying this infectious disease. Therefore, in the present study, we conducted a systematic review and meta-analysis to estimate the prevalence of BFDV infection, and to assess the associated risk factors and provide constructive and helpful measures and programmes to prevent and control the global spread of BFDV.

## 2. Materials and Methods

### 2.1. Search Strategy

The study was conducted according to the PRISMA guidelines (Preferred Reporting Items for Systematic Reviews and Meta-Analyses) [16]. The PRISMA checklist was used to ensure inclusion of all relevant information in the analysis (see Supplementary Materials Data S1).

Literature was retrieved from five databases: PubMed, Web of Science, Scopus, China National Knowledge Infrastructure (CNKI), and the WanFang database. The English PubMed search used was “Beak and Feather disease virus” OR “BFDV” OR “PBFDV” OR “PBFD” OR “circovirus” AND “prevalence” AND “psittaciformes” OR “psittacidae” AND ((“1 January 2000”[Date—Publication] : “31 December 2024”[Date—Publication])). Chinese search terms were “beak feather virus” or “BFDV” or “PBFDV” or “PBFD” or “circovirus” and “psittaciformes” or “psittacidae “and “prevalence”, published date limited from 1 January 2000 to 31 December 2024.

### 2.2. Study Inclusion and Exclusion Criteria

After removing duplicates and review articles, an initial screening was performed based on the title and abstract of each article to further exclude irrelevant studies that were not related to the prevalence of BFDV, such as studies on structural biology and whole genome sequencing. The full text was obtained and read to see if it met the following criteria: (1) the study population consisted of parrots; (2) the virus to be tested is beak and feather virus; (3) clearly defined time of sample collection and species, with random sampling applied; (4) clearly defined testing method; and (5) a cross-sectional study design. In the first step, titles and abstracts were screened for eligibility. Selection and identification of literature was performed independently by two evaluators. If the results were inconsistent, they were resolved by a third party or through discussion and negotiation.

### 2.3. Data Extraction

Literature that met the criteria for each item was independently screened by two researchers and specific information from the literature was individually extracted to prepare a data collection form in Microsoft Excel (2016 for Windows) software (Microsoft Corp., Redmond, WA, USA). The extracted data included: (1) Characteristics of the study: name of the paper, first author, date of publication of the paper; (2) methods of the study: time of sampling, place of sampling, type of samples, method of detection; (3) characteristics of the parrots: genera of parrots, sex, and age; and (4) prevalence (number of positive samples and total number of samples).

### 2.4. Literature Quality Assessment

As this study is not a randomized clinical trial, a standardized approach to its systematic evaluation has not yet been fully established. Therefore, based on the Cochrane quality assessment tool, combined with an improved critical appraisal tool (High Quality Item Rating Scale) developed by Munn et al. [17], we adapted the methodology for systematic evaluation in this study and assessed the risk of bias for the included studies. Quality assessment mapping was performed using RevMan 5.3 software, where the following seven items were examined and scored according to a simple scale: “Yes” was scored 2 and green, “Unsure” was scored 1 and yellow, and “No” was scored 0 and red. (1) Were the study animals recruited in an appropriate way? (e.g., wild-caught individuals were accompanied by detailed capture methods; donated specimens had clearly labeled sources.) (2) Was the study conducted in accordance with established animal welfare guidelines? (3) Was the origin of the animal clearly stated? (e.g., geographic information including countries or specific locations.) (4) Was the location of the study clearly stated? (5) Was the sampling period clearly stated? (6) Was the sampling method clearly stated? (e.g., PCR, hemagglutination (HA), or hemagglutination inhibition (HI)) (7) Were study animals categorized into different species or genera for statistical purposes?

### 2.5. Statistical Analysis

Data processing and statistical analysis were conducted using Stata 15.1 software (Stata Corp., College Station, TX, USA), and forest plots were analyzed using the metaprop packages [18]. The data were transformed using Freeman–Tukey double arcsine transformation. If the included studies reported multiple results from different diagnostic techniques for the same sample, the results from the more reliable PCR diagnostic technique were prioritized for use. If the included studies reported multiple results from different sampling methods, all these samples would be used.

### 2.6. Overall Pooled Prevalence of BFDV in Parrots

I^2^ and Q tests were used to evaluate whether the effect sizes of the studies were heterogeneous or not. *p* ≥ 0.10 and I^2^ < 50% indicated that there was low or no statistical heterogeneity among the effect sizes of the studies; therefore, they should be analyzed using a fixed-effects model [19]. *p* < 0.10 and I^2^ ≥ 50% indicated that there was large statistical heterogeneity between the effects of the studies; therefore, the Dersimonian–Laird random effects model (D-L method) should be used.

Although the inverse variance method is widely used and is suitable for 50% prevalence rates, when the rate is too small or large (0–30% or 70–100%), the research will be weighted more and other issues might arise; thus, such extreme values affect the final result and need to be adjusted to achieve a normal distribution as far as possible [20]. Therefore, before combining the prevalences, we converted the data using the Freeman–Tukey double arcsine transformation [21,22] as follows:

In Equation (1): t = PCR conversion positivity rate, r = number of positive samples, n = total number of samples. In Equation (2): se = standard error. Equation (3) was used to reverse the scale:

(1) t=arcsin⁡[sqrt{r/(n+1)}]+arcsin[sqrt{(r+1)/(n+1)}]

(2) $$se\left(t\right)=sqrt\left\{1/(n+0.5)\right\}$$

(3) $$p=\left(sin\left(\frac{t}{2}\right)\right)²$$

Heterogeneity of prevalence in the included studies was assessed by Q-test and I^2^ statistics. A *p*-value of less than 0.05 (from the Cochrane Q-test) indicated significant heterogeneity. I^2^ values of 25%, 50%, and 75% implied low, intermediate, and high levels of heterogeneity, respectively, and their 95% confidence intervals (CI) were calculated for each study; then, point estimates of the combined positivity rates of all the included studies were analyzed along with their 95% CI [23,24].

### 2.7. Subgroup Meta-Analysis of Heterogeneity

Potential sources of heterogeneity were further investigated by subgrouping the data according to relevant characteristics. In this study, subgroups were analyzed by genus, sex, age, and season.

### 2.8. Bias and Sensitivity Tests

This study analyzed the bias of the published literature using funnel plots and the Egger test. The Egger test was used to quantify publication bias using rank regression and linear regression methods, respectively, with *p* < 0.05 indicating the presence of significant publication bias and *p* ≥ 0.05 indicating a low risk of publication bias [25]. In asymmetric funnel plots, pruning was used to interpolate potentially missing studies and estimate the corrected prevalence.

## 3. Results

### 3.1. Literature Search and Study Characteristics

**Figure 1:**
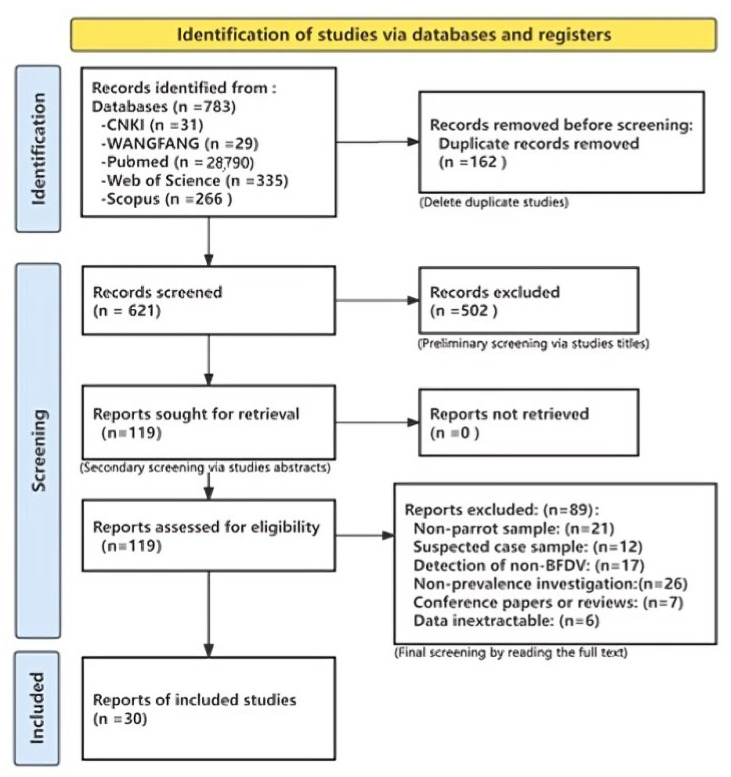
Flow diagram of the selection of eligible studies.

Based on the defined search strategy, five databases were searched and 783 papers published from 1 January 2000 to 31 December 2024 were collected. After excluding 162 cross-duplicates in the database, titles and abstracts were initially evaluated according to the Cochrane Handbook’s Literature Screening Process, and a further 621 irrelevant documents were excluded. The remaining 119 papers were screened a second time according to the inclusion and exclusion criteria, and ultimately, 30 papers were included for the meta-analysis. The specific process and results are shown in Figure 1.

The 30 studies were conducted between 1993 and 2022, and published between 2000 and 2024. The total number of parrots tested was 16,901, ranging from 12 to 4243 per study. PCR techniques were used in all 30 studies. The characteristics of the included studies are shown in Table 1.

### 3.2. Quality Appraisal of Included Studies

**Figure 2:**
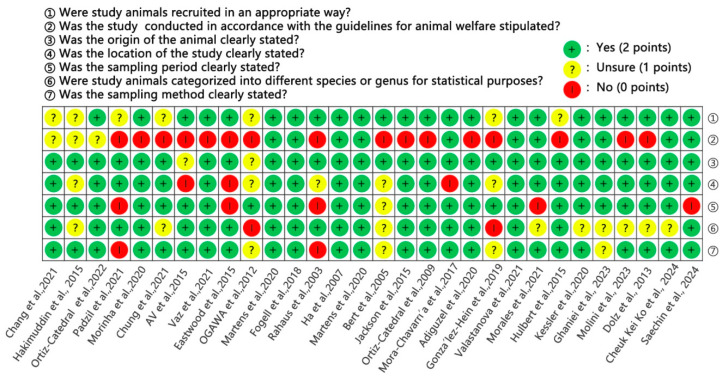
Quality evaluation of eligible studies [26,27,28,29,30,31,32,33,34,35,36,37,38,39,40,41,42,43,44,45,46,47,48,49,50,51,52,53,54].

According to the seven quality assessment items, the total possible score was 14 points. The scores of the papers included in the study ranged from 6 to 14. The median was 12, as shown in Figure 2.

### 3.3. Pooling and Heterogeneity Analyses

**Figure 3:**
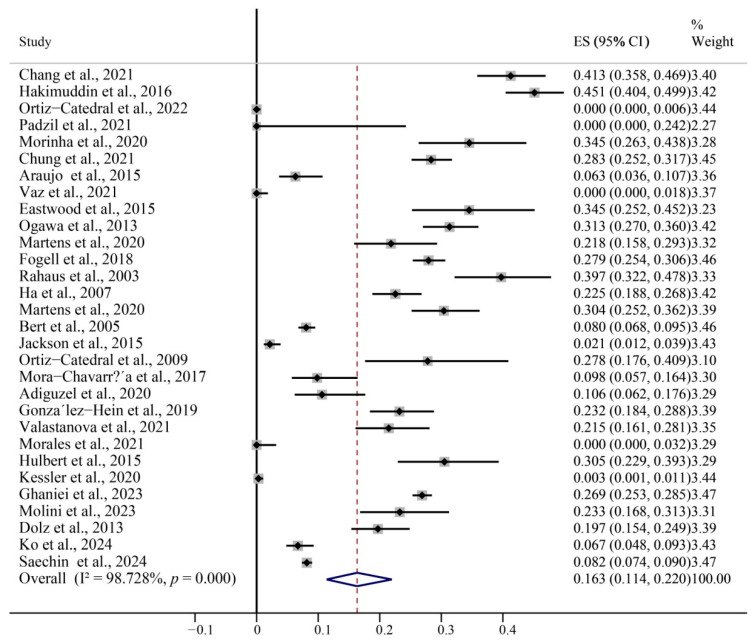
Forest plot of the prevalence of BFDV in parrots with random-effects analyses [26,27,28,29,30,31,32,33,34,35,36,37,38,39,40,41,42,43,44,45,46,47,48,49,50,51,52,53,54].

**Figure 4:**
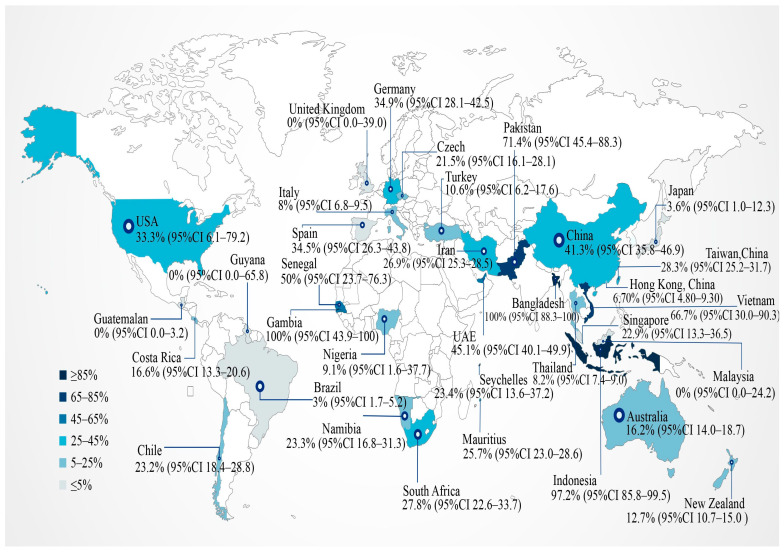
Prevalence of BFDV in parrots in different countries.

The global BFDV prevalence from 1993 to 2020 (published from 2003 to 2024) is shown in a forest plot (Figure 3). BFDV prevalence ranged from 0.00% to 45.13%, with significant heterogeneity among studies (χ^2^ = 2279.81; *p* < 0.01; I^2^ = 98.73%). Therefore, the pooled prevalence was calculated using a random effects model. The result was 16.30% (95% CI, 11.40–22.00%). The global molecular prevalence of BFDV in parrots is shown in Figure 4.

### 3.4. Subgroup Meta-Analysis for Prevalence Estimation

Subgroup analysis was carried out to identify the sources of heterogeneity and the results of the subgroup meta-analysis are shown in Table 2. The significantly high heterogeneity between subgroups in most of the studies meant that the pooled prevalence estimates for each subgroup were calculated using the random-effects Dersimonian–Laird model.

Subgroup analysis of sampling periods showed that the pooled prevalence of PBFD was 11.60% (95% CI, 4.00–22.20%) between 1993 and 2010, whereas it increased to 18.70% (95% CI, 10.70–28.20%) between 2010 and 2022, with no significant difference (*p* = 0.31) between the different sampling periods.

Subgroup analysis revealed significant differences in prevalence estimates across the four sampling methods (*p* < 0.01). Cloacal swabs showed the highest prevalence at 30.80% (95% CI, 23.10–39.00%), while blood samples had the lowest at 11.20% (95% CI, 4.40–20.70%). The prevalence for feather and fecal samples was 19.30% (95% CI, 13.20–26.30%) and 16.8% (95% CI, 6.00–31.20%), respectively.

The subgroup analysis by continent revealed highly significant variations in the prevalence of BFDV infection among parrots across different continents (*p* < 0.01). Africa exhibited the highest prevalence rate at 26.20% (95% CI, 23.90–28.50%), followed by Asia at 25.30% (95% CI, 15.40–36.60%), Oceania at 15.00% (95% CI, 4.10–30.80%), Europe at 11.90% (95% CI, 4.40–22.00%), South America at 2.80% (95% CI, 0.00–22.30%), and North America with the lowest prevalence rate at 0.00% (95% CI, 0.00–0.00%).

Significant differences in prevalence were observed between seasonal subgroups (*p* < 0.01), with BFDV having the highest prevalence of 22.60% (95% CI, 17.80–27.80%) in spring, followed by 12.10% in autumn (95% CI, 0.00–39.70%), 9.00% in winter (95% CI, 4.60–14.60%), and 3.80% in summer (95% CI, 0.10–11.10%).

There were significant differences (*p* = 0.04) in prevalence among different ages, with the highest prevalence of BFDV in young birds at 22.50% (95% CI, 8.80–39.60%), followed by nestling parrots at 10.30% (95% CI, 7.90–13.00%), and the lowest in adults at 4.50% (95% CI, 0.10–12.90%). No significant differences in prevalence were observed between the sexes (*p* = 0.96).

Subgroup analyses were conducted for different parrot genera based on data from studies with sample sizes greater than five. There were significant differences within parrot genera (*p* < 0.01). The *Agapornis* genus had the highest prevalence at 26.60% (95% CI, 9.80–46.50%) and the next genera were *Melopsittacus* with a prevalence of 22.60% (95% CI, 10.80–36.60%), *Trichoglossus* with 22.60% (95% CI, 6.10–43.80%), and *Poicephalus* with 21.40% (95% CI, 0.60–54.10%). The genus with the lowest prevalence was *Pionus* with 0.40% (95% CI, 0.00–12.70%).

### 3.5. Publication Bias and Sensitivity Analysis

**Figure 5:**
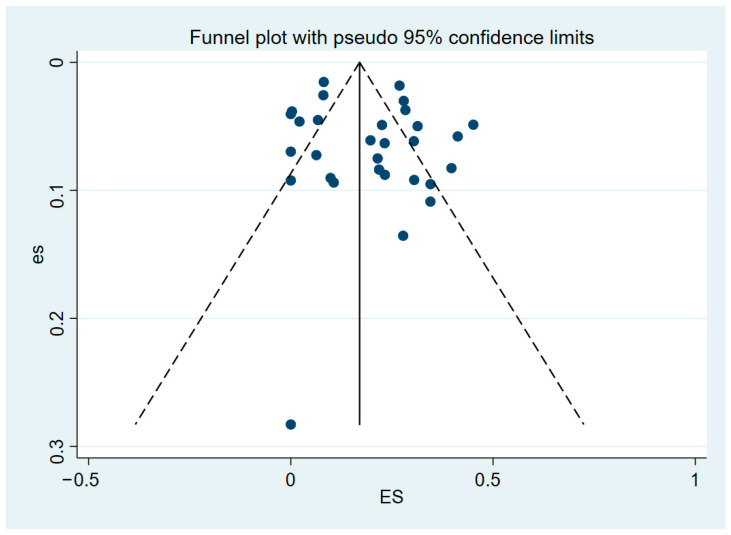
Funnel plot showing publication bias in studies reporting the prevalence of BFDV in parrots.

Sensitivity analyses showed that the point effect values for all indicators were within the 95% CI of the final effect value for overall prevalence. Some studies fell outside the confidence intervals, as seen in the funnel plot (Figure 5), indicating that the selected eligible studies had publication bias.

**Figure 6:**
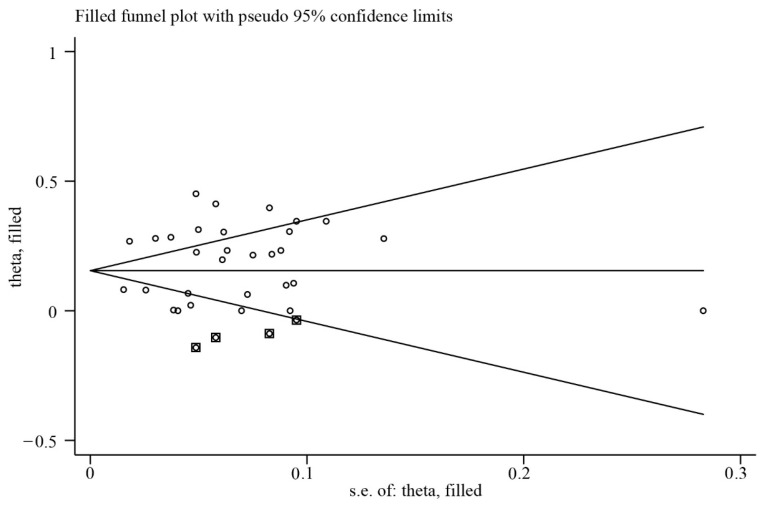
Funnel plot with trim and fill testing for publication bias (dot: the actual studies; box: the imputed missing studies).

Based on the assumption that publication bias leads to funnel graph asymmetry, an iterative method was used to estimate the number of missing studies, and a new meta-analysis was conducted using the shear compensation method (Metatrim command in Stata software) to determine the impact of publication bias on the research results. The Egger test was used to determine the publication bias among the selected literature (*p* < 0.05). Therefore, a trim and filling method was used to evaluate the influence of publication bias on the research results. After including four additional studies, the corrected bias was found to be not large, which showed that the combined results were robust (Figure 6).

## 4. Discussion

In the present study, a meta-analysis and systematic review were used to evaluate the pooled BFDV prevalence in parrots. A comprehensive analysis of papers published between 2003 and 2024 was conducted to determine the pooled prevalence of BFDV worldwide. Based on 30 eligible studies, the pooled prevalence was estimated at 16.30%.

BFDV has been found to be prevalent in most countries worldwide, posing a significant and worrying threat to the health of domesticated parrots, endangered parrots in the wild, other birds, and even other species [55,56,57]. The prevalence of BFDV cases is increasing markedly in some regions, with a recent report indicating a 24.48% prevalence of BFDV in Namibia [51]. Kasimov et al. [58] found that the prevalence rate of BFDV was the highest among all pathogens detected, reaching 33.54%, in 486 wild birds sampled across Southeast Queensland, Australia. BFDV is capable of infecting a wide range of hosts and can cross species barriers. It has also been detected in *Merops ornatus* (rainbow bee-eater) [59], *Zosterops simplex* (Swinhoe’s white-eye) [53], pigeons [60], and raptors such as *Ninox boobook* (southern boobook owl) [61] and *Erythrotriorchis radiatus* (red goshawk) [62]. A novel circovirus closely related to BFDV has even been identified in laboratory rabbits [63]. These findings highlight the urgent need for continuous global surveillance and the implementation of effective strategies to control the cross-species and cross-border spread of BFDV.

The prevalence of BFDV varies across different continents. In Australia and New Zealand, BFDV infection is widespread among parrot populations, with Australia being considered the origin of the pathogen [64]. Consequently, most research on BFDV has focused on isolates from Australia. However, in this study, the highest prevalence rates were observed in Asia and Africa. Therefore, we propose that insufficient resources dedicated to surveillance and control measures in regions such as Africa and Asia may play a crucial role in the widespread prevalence of viral infections [65]. The absence of robust surveillance systems and effective intervention strategies likely facilitates the rapid transmission of the virus among avian populations [66].

Our seasonal subgroup analysis indicated that spring and autumn are peak periods for BFDV prevalence. Warm and humid climates appear to enhance viral transmission [67], and increased bird activity, particularly during mating and nesting seasons, may further facilitate the spread of pathogens. These combined factors likely contribute to seasonal surges in BFDV outbreaks [68]. In contrast, the prevalence in summer was significantly lower, suggesting that hot and dry conditions may hinder BFDV transmission, consistent with the findings of A. Saechin et al. [54]. However, the data for spring were relatively limited, underscoring the need for future studies with a more extensive body of longitudinal data to clarify seasonal effects. Moreover, the observed seasonal trends are largely based on regions with four distinct seasons; in areas with only wet and dry seasons, the seasonal dynamics of BFDV remain to be clarified.

Our age subgroup analysis revealed that the infection rate in juvenile birds was significantly higher than that in adult birds. Michelle Wille et al. [69] reported that both viral abundance and diversity were marginally higher in juveniles compared to adults. PBFD tends to manifest when young birds experience stress from food shortages and breeding during feather development [31,70]. An additional contributing factor may be the immaturity of the immune system in juveniles, resulting in lower immunity [71]. Furthermore, it should be noted that BFDV comprises multiple strains, and birds at different developmental stages or of different species may be susceptible to distinct or evolved viral strains [15].

In this study, we compared the prevalence of BFDV across 19 genera within the family Psittacidae. The genera *Agapornis*, *Melopsittacus*, *Trichoglossus*, *Poicephalus*, and *Psittacula* all exhibited prevalence rates exceeding 20%, suggesting that parrots from these genera may be more susceptible to infection, resulting in higher disease incidence. *Agapornis* species, which are colorful, relatively inexpensive, and among the most commonly traded parrots in the pet market [72], may exhibit elevated BFDV prevalence due to frequent trade and transportation. The observed differences in BFDV prevalence among wild psittacine genera may partly be attributed to species-specific adaptations that allow certain hosts to coexist with the virus in the wild [70]. Genetic differences, immune responses, and interactions with other bird species may also contribute to variations in BFDV prevalence among different taxa [53,70,71]. The differences among varieties may be attributed to variations in the virulence of different BFDV strains [15]. A similar study by Patrizia et al. [73] found that African grey parrots are more susceptible to BFDV infection compared to other species and are more likely to develop severe clinical manifestations. These findings underscore the importance of prioritizing surveillance and control efforts toward species exhibiting high susceptibility to BFDV infection.

The gender subgroup analysis in this study showed no significant difference in infection rates between males and females, which is consistent with the findings of Martens et al. [15] and Blanch-Lázaro et al. [70]. Some studies have indicated that female parrots exhibit lower BFDV prevalence and viral loads compared to males [36]. Current evidence remains inconclusive, with no consistent pattern observed across studies.

Notable differences in detection outcomes were also observed among various sampling methods. This study found that BFDV prevalence was lowest in blood samples. Michael Hess et al. [74] reported similar results, noting reduced PCR sensitivity in blood samples compared to feathers and cloacal swabs. Likewise, a study by Blanch-Lázaro et al. [70] demonstrated that blood samples from adult parrots had lower detection rates than other tissues such as the liver and bone marrow. It has been reported that some adult birds are able to clear the virus from their bloodstream [36]. These findings suggest that blood samples, particularly from adult birds, may have limited diagnostic sensitivity [70]. This suggests that relying on blood samples for screening may lead to an underestimation of the true prevalence of BFDV. The differences in BFDV detection rates among sampling methods may be attributed to variations in viral load, patterns of viral shedding, or opportunities for environmental contamination. Therefore, we recommend that, where possible, multiple sampling methods should be employed to achieve a more accurate estimation of BFDV prevalence.

Diagnostic techniques, including various PCR assays, differ in their sensitivity and specificity. All included papers used PCR for BFDV detection, and most followed standardized sampling procedures, retesting protocols, and sensitivity analyses to reduce the influence of false-negative results. Compared to other methods, PCR offers superior sensitivity and specificity [75]. Nonetheless, it may still yield false-negative results [76]. Although detection techniques are continually being developed to improve diagnostic efficiency for BFDV [77,78], special caution is warranted when using blood samples, as they may increase the risk of false negatives. Therefore, during the trade and translocation of parrots, it is advisable to employ multiple diagnostic approaches and implement comprehensive population-level surveillance to enhance detection accuracy.

There are limitations to this study linked to the quality and quantity of some literature publications we relied upon. We selected seven potential risk factors for subgroup analyses. However, factors such as climatic variation across regions and insufficient data from specific countries may have introduced bias. The limited number of eligible studies in certain subgroups may have affected the robustness and completeness of the meta-analysis. Additionally, the possibility of false-negative results remains a notable limitation of PCR-based detection. Furthermore, this study did not include comparative analyses of urban captive, free-ranging, and wild populations, which limits our ability to assess the potential risk of BFDV mutation and evolution in captive parrots.

## 5. Conclusions

A comprehensive meta-analysis of 30 studies revealed a pooled global BFDV prevalence of 16.30%, exhibiting a gradual increase over time. BFDV prevalence was highest in Asia and Africa. Among the 19 psittacine genera analyzed, *Agapornis* was identified as being particularly susceptible to BFDV infection. Relying solely on blood testing may lead to an underestimation of true prevalence. Juvenile birds exhibited significantly higher infection rates than adults. Strengthened global surveillance of PBFD is recommended, particularly in resource-limited regions with inadequate disease monitoring infrastructure. To improve efficiency and cost-effectiveness, surveillance efforts should prioritize juvenile birds and highly susceptible species, while incorporating a range of tissue types to ensure accurate detection.

## Figures and Tables

**Table 1:** Characteristics of the eligible studies.

No	Authors	Study Years	Nation	Diagnostic Techniques	Sample Time	Sample Type	Sample Size	No. Positive
1	Chang et al., 2021 [26]	2021	Mainland China	PCR	2017–2018	③	298	123
2	Hakimuddin et al., 2016 [27]	2016	UAE	PCR	2009–2014	①/②	421	190
3	Ortiz-Catedral et al., 2022 [28]	2022	Australia	PCR	1997–2007, 1993–1995 , 1999, 2000, 2014	①	612	0
4	Padzil et al., 2021 [29]	2021	Malaysia	PCR	/	③	12	0
5	Morinha et al., 2020 [30]	2020	Spain	PCR	2015–2018	①	110	38
6	Chung et al., 2021 [31]	2021	Taiwan	PCR	2019	①	720	204
7	Araujo et al., 2015 [32]	2015	Brazilian	PCR	2009–2010	①/④	190	12
8	Vaz et al., 2021 [33]	2021	Brazil	PCR	2013–2018	①/④/⑤	205	0
9	Eastwood et al., 2015 [34]	2015	/	PCR	/	①/②/⑤	84	29
10	Ogawa et al., 2013 [35]	2013	Guyana, Indonesia, Japan, Singapore, South Africa, U.S.A.	PCR	2003–2004	①/②	402	126
11	Martens et al., 2020 [36]	2020	Australia	qPCR	2016–2018	①/④	142	31
12	Fogell et al., 2018 [37]	2018	Bangladesh, Gambia, Germany, Japan, Mauritius, Nigeria, Pakistan, Senegal, Seychelles, South Africa, United Kingdom, Vietnam, Western Africa	PCR	2013, 2014, 2015, 2013–2015, 2007–2010, 2015, 2009–2011, 1994–2016, 2009–2012	①/②/⑤	1103	308
13	Rahaus et al., 2003 [38]	2003	Germany	PCR	/	②	146	58
14	Ha et al., 2007 [39]	2007	New Zealand	PCR	2001–2004, 2004–2006	②	417	94
15	Martens et al., 2020 [15]	2020	Australia	PCR	2017–2018	①/④/⑤	263	80
16	Bert et al., 2005 [40]	2005	Italy	PCR	2000–2004	①	1516	122
17	Jackson et al., 2015 [41]	2015	New Zealand	PCR	2011–2013	①/②	467	10
18	Ortiz-Catedral et al., 2009 [42]	2009	New Zealand	PCR	2008	②	54	15
19	Mora-Chavarría et al., 2017 [43]	2017	Costa Rica	PCR	2011–2012	①/②	122	12
20	Adiguzel et al., 2020 [44]	2020	Turkey	PCR	2016–2020	③	113	12
21	González-Hein et al., 2019 [45]	2019	Chile	PCR	2013–2016	②	250	58
22	Valastanova et al., 2021 [46]	2021	Czech Republic	nested PCR	/	②	177	38
23	Morales et al., 2021 [47]	2021	Guatemala	real-time PCR	/	①	117	0
24	Hulbert et al., 2015 [48]	2015	Australia	PCR	/	③	118	36
25	Kessler et al., 2020 [49]	2020	Germany, France	PCR	2012–2017	①/④/⑤	680	2
26	Ghaniei et al., 2023 [50]	2023	Iran	PCR	2019–2021	②	3029	814
27	Molini et al., 2023 [51]	2023	Namibia	PCR	2021–2022	④	129	30
28	Dolz et al., 2013 [52]	2013	Costa Rica	PCR	2005–2009	③	269	53
29	Ko et al., 2024 [53]	2024	Taiwan, China	PCR	2019–2022	③	492	33
30	Saechin et al., 2024 [54]	2024	Thailand	PCR	2012–2017	①	4243	346

Abbreviations: PCR, polymerase chain reaction. ① blood; ② feathers; ③ fecal swabs; ④ cloacal swabs; ⑤ other tissue samples.

**Table 2:** Pooled estimates of BFDV in parrots by potential risk factors.

Group	No. Studies	Total No. of Parrots	No. of Positive Parrots	Prevalence		Heterogeneity			*p*-Value
				Estimates	(95% CI)	Q (x^2^)	PQ	I^2^ (%)	
Overall	30	16,901	2874	16.30%	11.40–22.00%	2279.81	<0.01	98.73%	
Continentsa									
Asia	10	9518	1813	25.30%	15.40–36.60%	953.62	<0.01	99.06%	<0.01
North America	2	120	1	0.00%	0.00–0.00%	NA	NA	NA	
Oceania	7	1949	221	15.00%	4.10–30.80%	424.02	<0.01	98.59%	
Africa	3	1389	365	26.20%	23.90–28.50%	1.09	0.58	0.00%	
South America	4	647	70	2.80%	0.00–22.30%	99.93	<0.01	97.00%	
Europe	10	3163	335	11.90%	4.40–22.00%	348.33	<0.01	97.42%	
Age									
Nestling	2	578	128	10.30%	7.90–13.00%	NA	NA	NA	0.04
Young	5	548	108	22.50%	8.80–39.60%	42.60	<0.01	90.61%	
Adult	7	959	77	4.50%	0.10–12.90%	104.38	<0.01	94.25%	
Sex									
Male	4	243	50	21.40%	5.70–43.00%	36.75	<0.01	91.84%	0.96
Female	4	215	54	20.90%	6.00–41.00%	30.27	<0.01	90.09%	
Season									
Spring	2	273	80	22.60%	17.80–27.80%	NA	NA	NA	<0.01
Summer	4	1796	118	3.80%	0.10–11.10%	43.67	<0.01	93.13%	
Autumn	4	420	87	12.10%	0.00–39.70%	126.39	<0.01	97.63%	
Winter	3	1360	116	9.00%	4.60–14.60%	8.22	0.02	75.67%	
Sampling years									
1993–2008	9	3495	424	11.60%	4.00–22.20%	440.11	<0.01	98.18%	0.31
2009–2022	16	7560	1691	18.70%	10.70–28.20%	1260.64	<0.01	98.81%	
Sampling methods									
blood	9	6911	821	11.20%	4.40–20.70%	826.50	<0.01	99.03%	<0.01
feather	9	5398	1225	19.30%	13.20–26.30%	192.61	<0.01	95.85%	
fecal	6	1302	257	16.80%	6.00–31.20%	160.67	<0.01	96.89%	
cloacal swabs	3	373	115	30.80%	23.10–39.00%	5.69	0.06	64.85%	
Genus									
Agapornis	9	234	43	26.60%	9.80–46.50%	33.77	<0.01	76.31%	<0.01
Amazona	12	931	53	8.00%	2.80–15.00%	88.13	<0.01	87.52%	
Ara	14	2081	100	4.20%	0.10–11.70%	80.89	<0.01	83.93%	
Aratinga	13	512	77	2.10%	0.00–12.30%	56.84	<0.01	78.89%	
Cacatua	10	1574	280	18.90%	9.30–30.30%	113.51	<0.01	92.07%	
Diopsittaca	5	69	6	1.20%	0.00–9.00%	2.23	0.69	92.07%	
Eclectus	8	589	45	4.60%	0.00–20.50%	98.48	<0.01	92.89%	
Eolophus	7	89	12	17.80%	0.90–43.70%	26.26	<0.01	77.16%	
Melopsittacus	10	372	78	22.60%	10.80–36.60%	59.37	<0.01	84.84%	
Myiopsitta	7	317	32	7.10%	0.00–25.20%	51.72	<0.01	88.40%	
Nymphicus	10	1496	590	10.10%	0.00–34.60%	207.20	<0.01	95.66%	
Pionites	6	276	13	8.30%	0.00–29.30%	30.61	<0.01	83.67%	
Pionus	8	56	4	0.40%	0.00–12.70%	9.59	0.21	27.04%	
Platycercus	11	594	128	12.90%	2.70–26.90%	77.22	<0.01	87.05%	
Poicephalus	6	199	55	21.40%	0.60–54.10%	73.94	<0.01	93.24%	
Psittacula	13	2375	530	20.20%	7.40–36.50%	639.72	<0.01	98.12%	
Psittacus	11	1361	267	15.00%	3.00–31.70%	347.38	<0.01	97.12%	
Pyrrhura	7	458	36	7.70%	0.00–26.00%	45.06	<0.01	86.68%	
Trichoglossus	7	155	32	22.60%	6.10–43.80%	25.88	<0.01	76.82%	

CI, confidence interval; NA, not applicable.

## Data Availability

Data are contained within the article.

## Supplementary Materials

The following supporting information can be downloaded at: https://www.mdpi.com/article/10.3390/ani15101473/s1, Data S1. PRISMA 2009 Checklist 1.
