# Supplementary material for: A Meta-Analysis of Global Prevalence of Psittacine Beak and Feather Disease Virus Infection and Associated Risk Factors
Source: Animals (Basel). 2025 May 20;15(10):1473. doi: 10.3390/ani15101473 (PMC12108240; doi:10.3390/ani15101473)
Supplement: Supplementary file 1 [file animals-15-01473-s001.zip › animals-3571542-supplementary.pdf]

PRISMA 2009 Checklist (Adapted for KIN 4400)

Checklist for the manuscript **"A Meta analysis of global prevalence of Psittacine beak and feather disease virus infection and associated risk factors"**

Section/topic	#	Checklist item	Reported on page #
**TITLE**			
Title	1	Identify the report as a literature review.	1
**ABSTRACT**			
Structured summary	2	Provide a structured summary including, as applicable: background; objectives; data sources; study eligibility criteria, participants, and interventions; study appraisal and synthesis methods; results; limitations; conclusions and implications of key findings;	1-2
**INTRODUCTION**			
Rationale	3	Describe the rationale for the review in the context of what is already known about your topic.	2
Objectives	4	Provide an explicit statement of questions being addressed with reference to participants, interventions, comparisons, outcomes, and study design (PICOS).	2
**METHODS**			
Eligibility criteria	5	Specify study characteristics (e.g., PICOS, length of follow-up) and report characteristics (e.g., years considered, language, publication status) used as criteria for eligibility, giving rationale.	2
Information sources	6	Describe all information sources (e.g., databases with dates of coverage) in the search and date last searched.	3
Search	7	Present full electronic search strategy for at least one database, including any limits used, such that it could be repeated.	3
Study selection	8	State the process for selecting studies (i.e., screening, eligibility).	4
Risk of bias in individual studies	9	Describe methods used for assessing risk of bias of individual studies (including specification of whether this was done at the study or outcome level).	4

PRISMA 2009 Checklist (Adapted for KIN 4400)

Section/topic	#	Checklist item	Reported on page #
Risk of bias across studies	10	Specify any assessment of risk of bias that may affect the cumulative evidence (e.g., publication bias, selective reporting within studies).	4
**RESULTS**			
Study selection	11	Give numbers of studies screened, assessed for eligibility, and included in the review, with reasons for exclusions at each stage, ideally with a flow diagram.	4-5
Study characteristics	12	For each study, present characteristics for which data were extracted (e.g., study size, PICOS, follow-up period) and provide the citations.	5-8
Synthesis of results of individual studies	13	For all outcomes considered (benefits or harms), present, for each study: (a) summary of results and (b) relationship to other studies under review (e.g. agreements or disagreements in methods, sampling, data collection or findings).	8-11
**DISCUSSION**			
Summary of evidence	14	Summarize the main findings including the strength of evidence for each main outcome; consider their relevance to key groups (e.g., healthcare providers, users, and policy makers).	12-13
Limitations	15	Discuss limitations at study and outcome level (e.g., risk of bias), and at review-level (e.g., incomplete retrieval of identified research, reporting bias).	13
**CONCLUSION**			
Conclusions	16	Provide a general interpretation of the results in the context of other evidence, and implications for future research.	13

Adapted from: Moher, D.; Liberati, A.; Tetzlaff, J.; Altman, D.G. Preferred Reporting Items for Systematic Reviews and Meta-Analyses: The PRISMA Statement. *Ann. Intern. Med.* **2009**, *151*, 4; reprinted in *Phys. Ther.* **2009**, *89*, 873–880. https://doi.org/10.1093/ptj/89.9.873.

For more information, visit: **www.prisma-statement.org**.
